# NEWS

**DOI:** 10.7189/jogh.03.010202

**Published:** 2013-06

## THE BILL AND MELINDA GATES FOUNDATION

• In his annual letter for 2013, Mr Bill Gates emphasized the importance of measurement to improving the human condition. He reflected upon the agreement signed in 2000 by the United Nations “...as one of the greatest successes in terms of using measurement to drive global change”. The Millennium Development Goals, supported by 189 nations, specified the year 2015 as a deadline for making measurable improvements across a set of crucial areas that included income, health and education. As an example, the concrete goal of reducing child mortality by two–thirds created a clear target by which to measure success or failure; and regular assessment of the progress, followed by further improvements, are important means to achieving such global targets, to which political commitment was made. (*Wall Street Journal*, 25 Jan 2013)

• In a joint statement released by the Bill & Melinda Gates Foundation and the Carlos Slim Foundation, the two of the world's wealthiest persons, Mr Bill Gates and Mr Carlos Slim, said that they planned to use their foundations' resources to promote research and the development of agricultural technology. This should increase farmers’ productivity and reduce hunger among the world’s poor people. (*Forbes*, 13 Feb 2013)

• During his visit to Ghana in March 2013, Mr Bill Gates reinforced his belief that the world must commit to wiping out the remaining cases of polio and finally eradicate the disease, despite squeezed aid budgets and violence that has been plaguing vaccination efforts in a few remaining endemic areas. In April, he announced in Abu Dhabi that his foundation will contribute US$ 1.8 billion to the Global Polio Eradication Initiative, a third of the total funds needed. (*AFP*, 26 Mar 2013)

• In March this year, the Department of Biotechnology (DBT) of India, its Biotechnology Industry Research Assistance Council (BIRAC), and the Bill & Melinda Gates Foundation launched “Grand Challenges India”. Through this partnership, the government and the foundation will co–fund projects that aim to harness Indian innovation and research to develop affordable and sustainable solutions that could improve health in India and around the world. Under the Memorandum of Understanding, signed last year, the DBT and the Gates Foundation have agreed to invest up to US$ 25 million each over five years in innovations in vaccines, drugs, agricultural products, and interventions related to malnutrition, family and child health. DBT and BIRAC join a global network of the Grand Challenges framework, along with USAID, Grand Challenges Canada, and Grand Challenges Brazil. They are all committed to seeking out and rewarding not only established researchers in science and technology, but also young investigators, entrepreneurs, and other innovators to help expand the pipeline of ideas to fight major global health problems. (*BMGF*, 04 Apr 2013)

• Five Japan–based pharmaceutical companies announced that they would be forming a public–private partnership with the Japanese government. The new fund, called the “Global Health Innovative Technology” (GHIT), should assist low and middle–income countries in their fight against infectious diseases. The fund will be composed of Takeda, Astellas, Daiichi–Sankyo, Eisai and Shionogi, also including the Bill & Melinda Gates Foundation and the Japanese government. They will be developing medicines, vaccines and diagnostics for poor countries. (*Japan Daily*, 8 Apr 2013)

## THE GAVI ALLIANCE

• In December 2012, the GAVI Alliance, in an unprecedented move, partnered up with communications giant *Vodafone* in a bid to increase vaccination rates in developing countries of Africa. The three–year partnership, making Vodafone the eighth member of the GAVI alliance, will aim to establish mobile technology as a primary means of surveillance and tracking in terms of vaccination. Some ways in which the system would operate include reminding mothers of their child’s vaccination by text messages, enabling appointments to be made through telephones, enabling health workers to access patients’ health records through their phones, and monitoring stocks to ensure availability. This initiative will help GAVI achieve its aim of vaccinating a quarter of a billion more children by 2015. A pilot project is currently being set up in Mozambique. (*The Guardian*, 18 Dec 2012)

• The GAVI Alliance and its partners plan to support rotavirus vaccination in at least 40 countries, covering 50 million children by this life–saving intervention by the end of 2015. In 2006, the first countries to receive GAVI rotavirus funding were mainly in Latin America. The support will now be extended to additional states, including 16 African countries, where more than 50% of all rotavirus deaths occur. Rotavirus is the leading cause of life–threatening diarrhoeal disease and it is estimated to cause nearly half a million deaths each year. (*Vaccine News Daily*, 18 Dec 2012)

• Following the reports that approximately 800 children in Europe have developed narcolepsy after immunization with the H1N1 swine flu vaccine Pandemrix, manufactured by GlaxoSmithKline, the use of Pandemrix has been restricted since July 2011 in people under the age of 20 across Europe. Understandably, experts from both pharmaceutical industry and academia are wary to confirm causal link before more evidence is obtained, because rare adverse reactions can quickly develop into vaccine scares that spiral out of all proportion. (*Reuters*, 22 Jan 2013)

• GSK plans to form a joint venture in India to produce a six–in–one vaccine that will protect children in poor against infectious diseases including polio. A 50–50 venture with India’s Biological E Ltd should develop a vaccine that would combine Glaxo’s injectable polio shot with a vaccine produced by Biological E that protects against five diseases, including diphtheria and tetanus. The joint venture will cover the development costs for the candidate vaccine, with subsequent development costs split equally. (*Bloomberg*, 28 Jan 2013)

• The GAVI Alliance aims to protect more than 180 000 girls in eight African and Asian countries from cervical cancer by funding immunization projects with vaccines from *Merck* and *GlaxoSmithKline*. Ghana, Kenya, Laos, Madagascar, Malawi, Niger, Sierra Leone and Tanzania should be the first countries to receive support for cervical cancer protection pilot projects. (*Reuters*, 03 Feb 2013)

## THE WORLD BANK

• Dr Timothy G. Evans has been appointed as the World Bank's new Director for Health, Nutrition and Population. Dr Evans is currently the Dean of the James P. Grant School of Public Health of BRAC University in Bangladesh; he previously served as Assistant Director General at the World Health Organization, heading the Evidence, Information, Research and Policy Clusters, where he oversaw the production of the annual World Health Report. Dr Evans is well known as a leader in advancing global health equity and health systems performance, through his work with the Rockefeller Foundation and the Harvard School of Public Health. He also contributed to the development of innovative partnerships, including the GAVI Alliance, INDEPTH and Health Metrics Networks, the Global Health Workforce Alliance and the World Alliance for Patient Safety. He earned his DPhil in agricultural economics at Oxford, and pursued medical and postgraduate studies at McMaster and Harvard Universities. (*The World Bank*, 28 Jan 2013)

• Development assistance for health increased only by 2.5% (to US$ 28.1 billion) in 2012, after expanding at an average growth rate of 11% per year between 2001 to 2010, according to a report by the Institute for Health Metrics and Evaluation (IHME). Increased spending by the GAVI Alliance and UNICEF made up for lower contributions from the US, France and Germany. Governmental contributions dropped 4.4% – the US cuts were 3.3%, France's 13.0% and Germany's 9.1%, while UK and Australia were the only two nations among the six largest bilateral donors that increased funding. (*Bloomberg*, 6 Feb 2013)

• In 2012, the World Health Assembly endorsed a new health goal: to reduce avoidable mortality from non–communicable diseases (NCDs) by 25% by 2025 (the “25 by 25” goal). Although clearly trying to keep the momentum after the UN General Assembly's political declaration on the prevention and control of NCDs in 2011, the obstacles to achieving this goal are great and largely under–studied, because they are highly politically sensitive. Today, chronic NCDs remain the least recognised group of conditions that threaten the future of human health. (*The Lancet*, 12 Feb 2013)

• International aid for health is measured in billions of dollars, but it is still only a fraction of what most recipient country governments need to spend on health care for their people. In 2010, India received US$ 775 million in development assistance in health (DAH). This amount included financial DAH from the World Bank (IDA and IBRD), Asian Development Bank, African Development Bank, Inter–American Development Bank, Global Fund to Fight AIDS, Tuberculosis and Malaria, the GAVI Alliance, and the Bill and Melinda Gates Foundation. However, this amount was just 1.6% of what the both central government and state governments spent jointly on public health. (*Times of India*, 25 Feb 2013)

• The biggest emerging markets are uniting to tackle under–development and currency volatility. During an annual summit in Durban, South Africa, they discussed plans to set up institutions that could challenge the current roles of the World Bank and International Monetary Fund. The leaders of the BRICS nations – Brazil, Russia, India, China and South Africa – are set to establish a new development bank. They also discussed generating foreign–currency reserves to help them buffer the balance of payments or currency crises. The BRICS nations, with combined foreign–currency reserves of US$ 4.4 trillion and 43% of the world’s population, are seeking greater influence in global finance to match their growing economic power. They have already called for an overhaul of the leadership of the World Bank and IMF, which were created in Bretton Woods, New Hampshire, in 1944, and they oppose the practice of the respective presidents being drawn from the US and Europe. (*Bloomberg*, 26 Mar 2013)

## UNITED NATIONS

• For the first time in almost five years, the UN Secretary–General Ban Ki–moon returned to his own office. He expressed enthusiasm for the renovated Secretariat building, said to be one of the cleanest and most eco–friendly buildings in the world. The original had long been known to lack the standards expected of modern buildings, with water leakages, asbestos and poor ventilation systems. The renovation project, worth US$ 2 billion, has ensured improved safety, accessibility and environmental performance. The building, in its landmark site over the East River in Manhattan, has maintained its original iconic façade and is one of several buildings undergoing changes. Mr. Ban stated his belief that in their new headquarters the UN could “better serve the world’s people”. (*UN News*, 17 Dec 2012)

• The World Trade Organisation is seeking a new leader to take the helm at the world’s leading trade body, as the current leader Pascal Lamy steps down in August 2013. The long selection process has already shortlisted nine candidates, five of them from emerging economies around the world. The changes expected from the new Director–General could have a huge impact on the WTO, which has been struggling in a recent financial climate. The work of the WTO was once crucial in building trade capacity and negotiating trade deals, but in recent times it spends an increasing amount of time settling disputes. Moreover, it has often been criticised for not making trade fair and accessible for emerging economies. (*The Guardian*, 13 Jan 2013)

• A recent poll carried out by the Better World Campaign showed that around 8 out of 10 voters believe it is in America’s best interests to continue to actively support the United Nations, with voters of all political parties recognising the contemporary relevance of the United Nations. When asked specifically about the World Health Organisation, voters overwhelmingly voted that it was important for the US to remain a member. The results showed that very few voters would support a cut in international aid and development funding, contrary to proposals put forth by members of the congress. These findings, alongside many others in the report, have important implications for US politicians by clarifying voter opinion on spending and America’s global involvement. (*UN Dispatch*, 16 Jan 2013)

• The exodus of Syrians fleeing two years of violent conflicts increased the number of refugees in neighbouring countries to more than a million, according to the United Nations Refugee Agency. The number has increased by around 420 000 this year, with around 7000 to 8000 Syrians leaving the country daily. More than half of them are children under the age of 11. Millions more people are displaced internally. The United Nations High Commissioner for Refugees, António Guterres, warned that the international humanitarian response is dangerously stretched. Around 330 000 Syrians have sought shelter in Lebanon, 320 000 in Jordan, 185 000 in Turkey, 105 000 in Iraq, 43 500 in Egypt and around 8000 across North Africa, with others having fled to Europe. (*New York Times*, 06 Mar 2013)

• The UN's latest development report stated that poverty reduction in the developing world was exceeding all expectations. The report quotes an “epochal global rebalancing”, in which higher growth rates in at least 40 poor countries are presently helping to lift hundreds of millions out of poverty and into a new “global middle class”. According to the report, “never in history have the living conditions and prospects of so many people changed so dramatically and so fast”. The report explains that the brighter global picture is the result of “international and national aid and development projects, investing in schools, health clinics, housing, infrastructure and improved access to water”. The UN also pointed to trade as being a key factor, presently improving conditions in Afghanistan, Ethiopia, Rwanda and Sierra Leone. These improvements have not been measured in the past, when poverty has only been defined in income terms, without taking into account health, education and living standards. The new measure – the Multidimensional Poverty Index (MPI) – includes ten indicators to calculate poverty: nutrition, child mortality, years of schooling and attendance, cooking fuel, water, sanitation, electricity assets and a covered floor. (*The Guardian*, 17 Mar 2013)

## UN AIDS AND THE GLOBAL FUND

• At the Global Fund to Fight AIDS, Tuberculosis and Malaria (Global Fund), the AIDS expert Dr Mark Dybul has been appointed as the new Executive Director. Mr Dybul helped found and later managed the implementation of the President's Emergency Plan for AIDS Relief (PEPFAR) from 2006 to 2009. His term follows a global financial crisis and a managerial turmoil at the organization, as several donors ended their contributions, while uncertainties due to corruption charges continue. (*Lancet Infectious Diseases*, 7 Jan 2013)

• A child in Mississippi was reported “cured” of HIV. Doctors treating the two and a half year old said that a “functional cure” meant that they tested negative for the virus, although it was expected to still be present in small concentrations in her body. The child stopped receiving care one year previously. She had received intensive treatment with three antiretroviral medications 30 hours after birth, which made the child’s viral load undetectable within a month. Moreover, a report has emerged from the Pasteur Institute in Paris regarding 70 adults who received early multi–therapy ART, which was continued for an average of three years before treatment was disrupted; fifteen of those patients now have no traces of HIV in their blood. It appears that early and intensive treatment limits the infection of CD4 cells and therefore reduces HIV's ability to persist in the body, preserves the immune response and reduces the diversity of the virus, all of which appear to facilitate control of the virus. Although this progress received massive media attention, it offers little to the majority of HIV sufferers, as it is rare for the virus to be detected at such an early stage. (*UNAIDS*, 04 Mar 2013)

• Health leaders from Africa and international agencies gathered in Swaziland to launch a new initiative against tuberculosis (TB), including TB among people living with HIV. Backed by several new investments worth more than US$ 120 million, the leaders signed the “Swaziland Statement”. This document commits them to accelerate progress against the two diseases in the next 1000 days. They should also work with Southern African Development Community (SADC) countries to achieve the international targets of cutting deaths from TB and HIV–associated TB by half by 2015, in comparison to 1990 levels. (*The Global Fund/ReliefWeb*, 20 Mar 2013)

• The latest reports on AIDS in sub–Saharan Africa, the epidemic’s long–term epicentre, are bringing good news. New HIV infections have declined by 25% since 2001, with AIDS–related deaths decreasing by 32% over the past 6 years, with expanded options for testing and treatment. After decades of pessimistic news about AIDS in Africa, there is more optimism about the control of the pandemic. Most of the measured improvements in AIDS in Africa resulted from cumulative, widespread behaviour change that reduced new HIV infections. (*Slate*, 28 Mar 2013)

• The Global Fund to Fight AIDS, Tuberculosis and Malaria has set the target of raising US$ 15 billion to help countries fight the three infectious diseases in the 2014–2016 period. The majority of the funds are likely to come from governments of the United States, France, Britain, Germany and Japan, ie, the five largest donors to the Global Fund. The Global Fund's assessment report indicates that a total need of US$ 87 billion is forecast for 2014–2016 period for their HIV/AIDS, tuberculosis and malaria programs world–wide. (*Xinhua*, 9 Apr 2013)

## UNICEF

• In December 2012, the latest report from Multilateral Organisation Performance Assessment Network (MOPAN) was released. Formed in 2012, this association of seventeen countries aims to assess organisational effectiveness of multilateral organisations. The evaluation, which was lead by donor countries Austria and Spain, found that UNICEF has made remarkable strategic improvements in the past three years. This assessment was based on a survey completed by individuals from MOPAN donor governments, national partners and peer organisations. They indicated that UNICEF’s greatest strength is strong working relationship with its partners, followed by its technical expertise and credibility, significant field presence and close proximity to local actors on the ground. A particular area requiring improvement was heavy and inflexible administrative processes, including slow release of funds to partners. The next MOPAN report is expected in 2015. (*UNICEF*, 31 Dec 2012)

• The births of nearly half the world’s children are not registered, which leaves them outside support and protection systems and uncounted in policy decisions. UNICEF supports the right of every child to be registered at birth, without discrimination, which is a critical first step towards safeguarding child's lifelong protection – by establishing an official identity, a recognized name and a nationality. However, 49% of children under the age of 5 are not registered world–wide. Children with no birth certificate don’t exist before the law, and are in danger of remaining on the margins of society, facing major challenges in accessing health care, education and social assistance. (*UNICEF*, 12 Feb 2013)

• The United Nations Children's Fund (UNICEF) announced that more than a quarter of children under the age of 5 worldwide are permanently “stunted” from malnutrition, leaving them physically and intellectually weak. This represents an unacceptable waste of human potential. Mr Anthony Lake, UNICEF's Executive Director, said that organized provision of vitamins and clean water and a focus on breastfeeding from birth could have helped some 165 million children to achieve normal development. However, their lack of proper nutrition makes them increasingly vulnerable to illness and premature death. The countries with the highest levels of stunted children are concentrated in sub–Saharan Africa and South Asia. (*Associated Press*, 15 Apr 2013)

• UNICEF has warned that it would become unable to provide “life–saving” aid to Syrian refugees in Jordan and other countries due to shortage of funds. The demands related to the conflicts in Syria are rising exponentially, and Ms Marixie Mercado, a spokeswoman for UNICEF, declared the agency “broke” in terms of their capacity to continue to support the refugees. (*BBC News*, 5 April 2013)

• According to UNICEF, WHO and USAID, child deaths from pneumonia and diarrhoea could be virtually eliminated by 2025. The new plan seeks an “integrated approach”, starting with standard ways to protect children from infections, but also adding the newer vaccines against pneumococcal bacteria and rotavirus. Dr Elizabeth Mason, the Director of Maternal, Newborn, Child and Adolescent Health at the WHO, said that the amount required to achieve this would be just over US$ 6 billion to 2025. These funds could come from national health budgets, better use of existing funds, and partners, such as the GAVI Alliance. A successful effort against the biggest killers of children will require co–ordinated effort in 15 interventions, from clean water and antibiotics to vaccines. (*Reuters*, 12 Apr 2013)

## WORLD HEALTH ORGANIZATION

• The World Health Organisation (WHO) has recently qualified dengue as the fastest–spreading tropical disease today. The organisation estimates that 50 million cases occur annually, but recent research suggests that the actual numbers may be twice as much. The disease, spread principally by the mosquito *Aedes aegypti*, is now present on every continent. Europe suffered its first outbreak since the 1920s in 2012, with about two thousand people affected in Spain. A specialist at the WHO's control of neglected tropical diseases department said that the mosquitoes most probably reached Europe through the importation of ornamental bamboos and second–hand tyres. Although vector control strategies have been put in place in seaports and airports, it can be quite difficult to detect mosquito eggs. With such a quick dissemination, it is not unlikely that the world may face a dengue pandemic in the future. Vaccines against dengue are still largely in the experimental stages and the most advanced one, developed by Sanofi, proved to be effective only in 30% of cases in a clinical trial. (*Reuters*, 16 Jan 2013)

• Dr Hiroshi Nakajima, a Japanese physician who led the World Health Organization from 1988 to 1998, has died in Poitiers, France, at the age of 84. The Western nations twice opposed his election as director general, arguing that he had not infused the agency with a clear sense of direction, and had let its budget and bureaucracy increase far beyond reasonable need. (*New York Times*, 28 Jan 2013)

• For the first time, the three international organizations focused on health, intellectual property and trade – WHO, WIPO, WTO – have pooled their expertise on a study of policies needed to advance medical and health technologies and to ensure they reach the people who need them. Mr Greg Perry, executive director of the Medicines Patent Pool, said that their joint report “...adds further weight to the idea of public–health oriented licensing as a key win for all stakeholders in the public health arena: from pharmaceutical companies, to generic companies, to – most importantly – people living with HIV”. (*WHO*, 5 Feb 2013)

• The global development assistance for health (DAH) has increased by nearly 500% in the last two decades, but the funds disbursed through the World Health Organisation (WHO) increased by only 62%. The WHO's share of the global DAH fell from 25% to only 9% between 1990 and 2010, as revealed in a paper published in the *The Lancet* and authored by several employees of the WHO. The channelling of available resources through many new big players in global health has raised questions about the focus of health spending and the role of WHO. WHO's core budget is still mainly intended for providing leadership on crucial health issues, shaping the research agenda, setting norms and standards, promoting and monitoring implementation and assessing health trends. (*Times of India*, 11 Feb 2013)

• The Global Vaccine Action Plan (GVAP) for the period 2011–2020 has been endorsed by the 194 Member States of the World Health Assembly in May 2012. GVAP is a roadmap to prevent millions of deaths by 2020 through more equitable access to existing vaccines. The dimensions of equity that will require consideration include disparities between countries, adolescent and adult immunization, and immunization during emergencies. Historically, it took decades before vaccines used in high–income countries became available in low– and middle–income countries, and GVAP should address this inequity. (*WHO*, 1 Mar 2013)

**Contributors:**

Rachel Banfield, Frances Barclay, Vijna Hiteshna Boodhoo, Katherine Booth, Rachel Burge, Michael Charteris, Alexander Fullbrook, Jenny Hall, Ewan D. Kennedy, Nethmee S. Mallawaarachchi, Diana Rudan, Victoria Stanford, Goran Zangana

